# Quality of Life Assessment in Patients with Knee Osteoarthritis

**DOI:** 10.1192/j.eurpsy.2024.870

**Published:** 2024-08-27

**Authors:** A. Feki, I. Sellami, F. Ellouze, M. Yahya, S. Baklouti, M. H. Elleuch

**Affiliations:** ^1^Rheumatology; ^2^Occupational medicine, Hedi Chaker Hospital; ^3^Physical Medicine and Functional Rehabilitation, Habib Bourguiba Hospital, Sfax, Tunisia

## Abstract

**Introduction:**

Osteoarthritis is one of the most common diseases and a leading cause of functional limitation and dependence, significantly impacting the quality of life (QOL).

**Objectives:**

The aim of this study was to evaluate the impact of knee osteoarthritis on QOL and identify associated factors.

**Methods:**

This prospective cross-sectional descriptive study was conducted in the Physical Medicine and Functional Rehabilitation Department over a 4-month period, involving patients with symptomatic bilateral knee osteoarthritis (according to the American College of Rheumatology (ACR) criteria). Sociodemographic data, comorbidities, and characteristics of knee osteoarthritis were collected. The assessment of QOL and the functional impact of knee osteoarthritis were based on the KOOS (Knee Injury and Osteoarthritis Outcome Score) self-questionnaire, Lequesne Index, and modified WOMAC (Western Ontario and McMaster Universities Osteoarthritis Index) Score. The KOOS questionnaire included 5 subscales: pain (KOOS-Pain), symptoms other than pain (KOOS-Symptoms), activities of daily living (KOOS-ADL), sports and recreational function (KOOS-Sport), and QOL (KOOS-QOL).

**Results:**

We included 30 patients with an average age of 59.27±6.3; the male-to-female ratio was 0.15. Sixty percent of patients lived in urban areas, with varying levels of education: primary (n=10), secondary (n=4), and university (n=4), while the majority were illiterate (40%). Most of our patients were employed, with 64.28% engaging in significant physical activity, resulting in an average of 6±2 days of work absenteeism every 3 months due to knee pain. The mean duration of knee osteoarthritis was 7.97 years±3.14. The average pain visual analog scale (VAS) score was 5.2±0.4. Knee osteoarthritis was classified as stage 2 in 40% and stage 3 in 60% of cases. Regarding functional impact, the mean WOMAC global index was 16.6±4.68, and the mean Lequesne Index was 11.05±3.45; moderate disability was observed in 16.7%, significant disability in 50%, and severe disability in 16.7% of patients. Furthermore, the KOOS questionnaire revealed decreased KOOS-Sport and KOOS-QOL scores, with mean values of 35±10.2 and 37±8.9, respectively. Our study identified factors associated with a poor quality of life: age > 65 years (p<0.05), disease duration (p=0.02), and VAS pain > 5 (p=0.02).

**Conclusions:**

Improving the quality of life is an essential therapeutic goal in managing knee osteoarthritis. Our study demonstrates that advanced age, longer disease duration, and high pain intensity can negatively impact quality of life.

**Disclosure of Interest:**

None Declared

